# Self-reported fatigue following intensive care of chronically critically ill patients: a prospective cohort study

**DOI:** 10.1186/s40560-018-0295-7

**Published:** 2018-05-02

**Authors:** Gloria-Beatrice Wintermann, Jenny Rosendahl, Kerstin Weidner, Bernhard Strauß, Andreas Hinz, Katja Petrowski

**Affiliations:** 1 Department of Psychotherapy and Psychosomatic Medicine, Medizinische Fakultät Carl Gustav Carus, Technische Universität Dresden, Dresden Fetscherstraße 74, 01307 Dresden, Germany; 2 Center for Sepsis Control and Care, Jena University Hospital, Friedrich-Schiller University, Jena, Germany; 3 Institute of Psychosocial Medicine and Psychotherapy, Jena University Hospital, Friedrich-Schiller University, Jena, Germany; 4 Department of Medical Psychology and Medical Sociology, University of Leipzig, Leipzig, Germany

**Keywords:** Fatigue, Multidimensional Fatigue Inventory (MFI-20), Intensive care unit (ICU), Chronic critical illness (CCI), Sepsis, Health-related quality of life, Posttraumatic stress

## Abstract

**Background:**

Protracted treatment on intensive care unit (ICU) sets the patients at increased risk for the development of chronic critical illness (CCI). Muscular and cardio-respiratory deconditioning are common long-term sequelae, going along with a state of chronic fatigue. At present, findings regarding the frequency, long-term course, and associated factors of self-reported fatigue following ICU treatment of CCI patients are lacking.

**Methods:**

CCI patients with the diagnosis of critical illness polyneuropathy/myopathy (CIP/CIM) were assessed at three time points. Four weeks following the discharge from ICU at acute care hospital (t1), eligibility for study participation was asserted. Self-reported fatigue was measured using the Multidimensional Fatigue Inventory (MFI-20) via telephone contact at 3 (t2, *n* = 113) and 6 months (t3, *n* = 91) following discharge from ICU at acute care hospital.

**Results:**

At both 3 and 6 months, nearly every second CCI patient showed clinically relevant fatigue symptoms (t2/t3: *n* = 53/*n* = 51, point prevalence rates: 46.9%/45.1%). While total fatigue scores remained stable in the whole sample, female patients showed a decrease from 3 to 6 months. The presence of a coronary heart disease, the perceived fear of dying at acute care ICU, a diagnosis of major depression, and the perceived social support were confirmed as significant correlates of fatigue at 3 months. At 6 months, male gender, the number of medical comorbidities, a diagnosis of major depression, and a prior history of anxiety disorder could be identified. A negative impact of fatigue on the perceived health-related quality of life could be ascertained.

**Conclusions:**

Nearly every second CCI patient showed fatigue symptoms up to 6 months post-ICU. Patients at risk should be informed about fatigue, and appropriate treatment options should be offered to them.

**Trial registration:**

The present study was registered retrospectively at the German Clinical Trials Register (date of registration: 13th of December 2011; registration number: DRKS00003386). Date of enrolment of the first participant to the present trial: 09th of November 2011.

**Electronic supplementary material:**

The online version of this article (10.1186/s40560-018-0295-7) contains supplementary material, which is available to authorized users.

## Background

Subjective fatigue refers to an overwhelming, sustained sense of physical, emotional, and/or cognitive exhaustion that is not proportional to recent activity [1–3]. Fatigue has been most frequently related to cancer, its treatment, and chronic illnesses [4–7]. Beyond that, current research revealed that fatigue is one of the most prevalent and debilitating problem, emerging during critical illness and following the treatment on intensive care unit (ICU) [8–10].

Particularly, the long-term stay on ICU (> 72 h) along with prolonged mechanical ventilation may lead to a state of muscular/cardio-respiratory deconditioning, increasing the risk for chronic fatigue [11, 12]. Between 5 and 10% of acutely ill patients who require ongoing mechanical ventilation develop a syndrome named chronic critical illness (CCI) [13–15]. This comprises distinct clinical features (e.g., myopathy, neuropathy, loss of lean body mass, anasarca, vulnerability to infection and sepsis, delirium, coma). Characteristical is that CCI patients are not expected to be weaned from the ventilator in the immediate future, necessitating ongoing, tight cardio-pulmonary monitoring and long-term ventilator dependency [14]. As long-term sequelae, CCI patients might suffer from a chronic state of exhaustion which may interfere with the patient’s usual functional capacity and ability to participate in one’s own rehabilitation [16, 17].

At present, there is only preliminary evidence for the occurrence of fatigue in CCI patients [18–20]. In long-term treated ICU patients, chronic fatigue affected between more than one third and three quarter within 1 year after ICU discharge [18, 21, 22]. In line, Puntillo et al. showed that 75% of patients at high risk of dying reported “being tired” after prolonged ICU treatment [19]. However, the studies mentioned above did not apply either multidimensional and valid measures of fatigue, used short time frames, small sample sizes, or quite heterogeneous ICU samples.

Considering the etiopathogenetic mechanisms of fatigue, a multifactorial approach has been proclaimed, assuming the influence of sociodemographic, pathophysiological, and environmental factors, the impact of therapeutic interventions, and medication [16]. Above, fatigue goes along with emotional stress, a decreased health-related quality of life [23], and greatly overlaps with symptoms of depression, anxiety, and disordered sleep [16, 24–26]. However, there is still a need for studies unraveling subjective fatigue in CCI patients, especially its related factors [16]. This is of clinical relevance since an early identification would allow the referral of affected patients to appropriate symptom management programs. Therefore, the main aims of the present study were the following: first, the assessment of the rate and course of self-reported fatigue using the Multidimensional Fatigue Inventory (MFI-20) [27] at 3 and 6 months following ICU discharge; second, the identification of associated variables; and third, the impact of fatigue on psychological outcomes (e.g. posttraumatic stress, health-related quality of life) in CCI patients.

## Methods

### Setting and procedure

A homogeneous sample of patients with a principal diagnosis of critical illness polyneuropathy (CIP), critical illness myopathy (CIM), or combined CIP/CIM was consecutively enrolled during its treatment at a large rehabilitation hospital between November 2011 and May 2013. CIP and CIM have been shown to be important causes of the ICU-acquired weakness and failed weaning from the ventilator (e.g., [28]). The patients were assessed at three time points within an observational, prospective cohort study. The patient’s informed consent for study participation was obtained within 4 weeks following the transfer from ICU at acute care hospital to post-acute ICU at a rehabilitation hospital (t1). Subsequently, the patient’s cognitive status was assessed using the Confusion Assessment Method for the Intensive Care Unit (CAM-ICU; [29, 30]) vis-à-vis at bedside. The diagnosis of a delirium or a positive evaluation of the two CAM-ICU subtasks disorganized thinking and attention screening examination precluded the study participation and necessitated the repetition of the CAM-ICU after 2 weeks. In case that patients were not able to adequately communicate, their next-of-kin or designated power of attorney for health care was contacted to get the informed consent. At t2, 3 months following the transfer from acute care ICU to post-acute ICU, and at t3, 6 months post-transfer, the intensity of fatigue symptoms, posttraumatic stress symptoms, and the health-related quality of life were assessed using questionnaires via a telephone contact.

The present report was nested within a prospective-longitudinal cohort study with the primary goal to assess the rate and predictors of stress disorders following prolonged critical illness [31].

### Participants

Patients with a principal diagnosis of CIP (ICD-10: G62.80), CIM (ICD-10: G72.80), or CIP/CIM with or without sepsis were eligible for a study participation. Patients had to fulfill the following further inclusion criteria: age between 18 and 72 years, a minimum length of ICU stay of 6 days, mechanical ventilation, sufficient German language skills, informed consent, and absence of a current delirium. Above, data on the intensity of fatigue symptoms had to be available at 3 months following the discharge from ICU at acute care hospital. Participants who were not alert showed fluctuating attention or consciousness or could not communicate because of sedation or blocked tracheal cannula were not included.

### Measures

The *Confusion Assessment Method for the Intensive Care Unit* (CAM-ICU) [29, 30] is an instrument for the assessment of delirium in the ICU. In the present study, it was applied in order to screen the patients for the presence of acute cognitive dysfunctions. The number of correctly identified letters or pictures in the auditory or visual components of the Attention Screening Examination and the number of right answers in the subtask disorganized thinking were summed up to an achievement score.

The *Multidimensional Fatigue Inventory-20* (MFI-20) [23, 27] is a 20-item self-report measurement of fatigue severity. The MFI-20 was applied via telephone interview at 3 (t2) and 6 months (t3) following the discharge from acute care ICU. It covers the five dimensions General Fatigue, Physical Fatigue, Mental Fatigue, Reduced Motivation, and Reduced Activity. Items are summed up to a simple total score with a minimum value of 4 (absence of fatigue) and a maximum value of 20 for each subscale. A total fatigue score is calculated as the sum of the subscale scores (range 20–100). Higher total scores indicate higher levels of fatigue. Validity has been shown for different participant populations, e.g., cancer patients, army recruits, and chronic fatigue syndrome [27]. Internal consistency has been shown to be good for the General, Physical, and Mental Fatigue dimensions (Cronbach’s alpha .84) and adequate for the subscales Reduced Activity and Reduced Motivation (Cronbach’s alpha > .65) [27]. In the present study, Cronbach’s *α* was .91 at 3 and .93 at 6 months. The 75th percentile was chosen as cutoff value for high fatigue based on the subscale General Fatigue and the total score (53+) of the MFI-20 total score [23, 32, 33].

Additionally, a current diagnosis of an affective disorder was assessed by an experienced clinical psychologist using the *Structured Clinical Interview for the Diagnostic and Statistical Manual of Mental Disorders DSM-IV* (SCID) [34] at 3 months (t2) and 6 months (t3) following the discharge from ICU at acute care hospital. The assessment of a lifetime history of an affective disorder was only realized at 6 months.

The *Posttraumatic Stress Syndrome Scale* (PTSS-10) [35, 36] is a 10-item self-report questionnaire for the estimation of the intensity of posttraumatic stress symptoms (e.g., sleep disturbance, nightmares, frequent changes in mood) at 3 and 6 months following the discharge from acute care ICU. The total score is received by summing up the scores of all items (range 10–70). The internal consistency and test-retest reliability of the PTSS-10 can be regarded as high (Cronbach’s *α* = .92, test-retest reliability *r* = .89) [37]. In the present study, Cronbach’s *α* was .82 at 3 and .87 at 6 months.

The *Multidimensional Scale of Perceived Social Support* (MSPSS) [38] consists of 12 items and was applied to assess the perceived support from three social sources (family members, friends, significant others) at 3 and 6 months post-ICU. Scores of all items are summed up to a total score (range 12–84). Internal reliability has been shown to be high (Cronbach’s *α* .89–.93) [39]. In the present study, Cronbach’s *α* was .90 at t2 and .89 at t3.

The health-related quality of life was measured with the questionnaire *Euro-Quality of Life* (EQ-5D-3L) [40] at 3 and 6 months. The EQ-5D-3L assesses five dimensions (mobility, self-care, usual activities, pain/discomfort, and anxiety/depression). Additionally, the subjective state of health is assessed by a visual analogue scale (VAS) ranging from 0 (worst) to 100 (best). A single one-dimensional index value was generated based on a simple sum score according to Hinz et al. [41]. In the present study, Cronbach’s *α* for health-related quality of life was .74 at t2 and .75 at t3.

*Sociodemographic characteristics* (e.g., age, marital status, education), *medical history* (e.g., sepsis, location of sepsis, number of sepsis episodes, duration of invasive ventilation, duration of ICU stay, presence of somatic comorbidities, severity of medical illness), and a life-time diagnosis of a psychological disorder were obtained from the patient records at t1. The *Barthel index* (*BI*) was applied at admission and discharge from post-acute ICU by a trained study nurse. The BI is a measure of performance in activities of daily living in 11 domains (e.g., fecal/urinary incontinence) with values ranging between 0 and 100. Additionally, the early rehabilitation BI (range − 325–0) was used assessing seven further domains (e.g., intensive care supervision, tracheostomy tube management, mechanical ventilation, confusion, severe impairment of communication, dysphagia) [42]. The scores of both Barthel scales were summed up, yielding a minimum value of − 325 and a maximum value of 100. A higher value indicates a better performance. Interrater reliability is very high (*r* = .95). Test-retest reliability is good as well (*r* = .89) [43].

### Statistical methods

For normally distributed data, arithmetic means, and standard deviations, otherwise, medians and interquartile ranges are reported. For categorical variables, frequencies and percents are shown. Bivariate correlational analyses were calculated depending on the level of measurement between sociodemographic, clinical, and psychological variables and fatigue symptoms at t2 and t3. The course of fatigue symptoms was examined with the analysis of covariance (ANCOVA) for repeated measures for normally distributed subscales of the MFI-20 (General Fatigue) and the total fatigue score. As covariates, age and gender were included. In case of non-normally distributed subscales of the MFI-20, the sign test was applied. A missing value in one item of the MFI at t3 was recorded in one participant and replaced by the median of the subscale.

MFI-20 scores were compared with age- and gender-stratified subgroups of a normative German sample [23]. Standardized mean differences (Hedges’ *g*) with 95% confidence interval (CI) were calculated for these comparisons. As the cutoff value for high fatigue, the 75th percentile (53+) was used according to Kuhnt et al. [32]. For the classification with respect to high vs. low General Fatigue, the age- and gender-adjusted 75th percentiles from a German representative community sample were applied [23, 33].

Clinical (e.g., sepsis-related characteristics, length of mechanical ventilation/ICU stay, severity of medical illness/Barthel index, number of medical comorbidities), psychological (e.g., prior psychiatric history, diagnosis of PTSD/major depression, perceived fear of dying/helplessness at ICU), and sociodemographic (e.g., age, gender, family/education status) factors which were correlated with the dependent variable (MFI-20 total score) with a *p* value < .2 [44] were entered in a multivariable linear regression analysis. Correlational analyses using Spearman’s rank correlation were calculated between the MFI-20 total score, the health-related quality of life (EQ-5D-3L), and posttraumatic stress symptomatology (PTSS-10). For all analyses, a significance level of *α* = 0.05 (two-sided) was applied. All data were analyzed using SPSS 24 (SPSS Inc., Chicago, IL, USA).

## Results

### Descriptive data

**Fig. 1:**
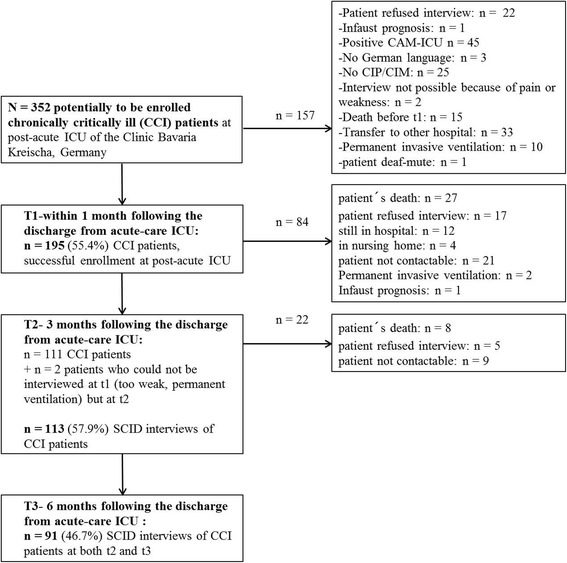
Flow chart including the dropped out patients and final sample of CCI patients. CAM-ICU: Confusion Assessment Method for the Intensive Care Unit; CIP/CIM: Critical Illness Polyneuropathy/Critical Illness Myopathy; SCID: Structured Clinical Interview for DSM (Diagnostic and Statistical Manual of Mental Disorders)–IV disorders

Of the *N* = 352 potentially to be enrolled CCI patients with the primary diagnosis of CIP, CIM or CIP/CIM, *n* = 157 (44.6%) patients could not be enrolled for different reasons (e.g., death, positive CAM-ICU, refusal of study participation; see Fig. 1). Finally, data of *n* = 113 patients were available at t2 and of *n* = 91 patients at t3 (Fig. 1). Table 1 displays the major sociodemographic, clinical, and psychological characteristics of the sample at 3 months (t2). CCI patients with complete data of the MFI-20 (*n* = 113) had a median age of 61.1 years. 72.6% (*n* = 82) were men. Acute respiratory insufficiency (*n* = 87, 77.0%), left heart failure (*n* = 40, 35.4%), and diabetes (*n* = 43, 38.1%) occurred as the most common medical comorbidities (Additional file 1: Table S1). Non-participants were significantly more severely ill than patients being followed up as shown by a lower Barthel index at discharge from post-acute ICU/rehabilitation hospital (data not shown, *p* < .001). Non-participants were less often educated 10 years or longer (non-participants vs. participants: 37.7 vs. 63.7%, *p* = .024). Above, significantly more non-participants suffered from hypertension (non-participants vs. participants: 21.8 vs. 12.4%, *p* = .036), organic brain syndrome (53.1 vs. 38.9%, *p* = .013), neurological disorders (37.7 vs. 23.9%, *p* = .010), or pneumonia (31.4 vs. 17.7%, *p* = .007).

**Table 1:** Descriptive characteristics of chronically critically ill (CCI) patients (*n* = 113) and the subsamples of patients with high (*n* = 61) vs. low fatigue (*n* = 52) at 3 months (t2) following the discharge from ICU at acute care hospital

Characteristic	Patients (*n* = 113)	High fatigue (*n* = 61)^a^	Low fatigue (*n* = 52)^a^	*U*/*χ*^2^ (*p*)^b^
Sociodemographic variables				
Age, years median (IQR)	61.1 (55.7–65.6)	61.5 (56.3–65.6)	58.7 (54.6–65.5)	1351.000 (.176)^c^
Gender, *n* (%)				
Male	82 (72.6)	45 (73.8)	37 (71.2)	
Female	31 (27.4)	16 (26.2)	15 (28.8)	.097 (.756)^d^
Family status, *n* (%)				
Single	10 (8.8)	5 (8.2)	5 (9.6)	
Married/cohabited	78 (69.0)	48 (78.7)	30 (57.7)	
Divorced/living apart	16 (14.2)	3 (4.9)	13 (25.0)	
Widowed	9 (8.0)	5 (8.2)	4 (7.7)	10.594 (.032*)^d^
Partnership				
Yes	78 (69.0)	48 (78.7)	30 (57.5)	
No	35 (31.0)	13 (21.3)	22 (42.3)	5.788 (.016*)^d^
Education, *n* (%)^e^				
< 10 years	35 (31.0)	17 (27.9)	33 (63.5)	
≥ 10 years	72 (63.7)	39 (63.9)	18 (34.6)	.296 (.587)^d^
Clinical variables				
Sepsis, *n* (%)				
No sepsis	36 (31.9)	18 (29.5)	18 (34.6)	
Sepsis	42 (37.2)	26 (42.6)	16 (30.8)	
Severe sepsis or septic shock	35 (31.0)	17 (27.9)	18 (34.6)	1.704 (.427)^d^
Number of sepsis episodes, median (IQR)	1.0 (0.0–1.0)	1.0 (0.0–1.0)	1.0 (0.0–1.0)	1512.500 (.911)^d^
Site of infection, *n* (%)				
Respiratory	56 (49.6)	32 (52.5)	24 (46.2)	.446 (.504)^d^
Urinary/genitals	12 (10.6)	5 (8.2)	7 (13.5)	.820 (.365)^d^
Abdominal	10 (8.8)	4 (6.6)	6 (11.5)	.863 (.509)^f^
Bones/soft tissue	6 (5.3)	4 (6.6)	2 (3.8)	.410 (.685)^f^
Wound infection	2 (1.8)	1 (1.6)	1 (1.9)	.013 (1.000)^f^
Heart	1 (.9)	1 (1.6)	0 (0.0)	.860 (1.000)^f^
Multiple	13 (11.5)	5 (8.2)	8 (15.4)	1.425 (.233)^d^
Others^g^	8 (7.1)	2 (3.3)	6 (11.5)	2.911 (.140)^f^
Unknown	4 (3.5)	2 (3.3)	2 (3.8)	.026 (1.000)^f^
Barthel index, median (IQR)				
At admission at post-acute ICU	− 200.0 (− 225.0–125.0)	− 185.0 (− 225.0–100.0)	− 200.0 (− 225–128.8)	1538.500 (.781)^c^
At discharge from post-acute ICU	− 35.0 (− 82.5–7.5)	− 25.0 (− 80.0–35.0)	− 40.0 (− 85.0–0.0)	1319.500 (.124)^c^
At discharge from rehabilitation hospital	65.0 (35.0–85.0)	65.0 (0.0–80.0)	75.0 (60.0–88.8)	1226.500 (.038*)^c^
ICU stay, days median (IQR)	66.0 (49.0–93.5)	69.0 (46.0–87.0)	62.0 (49.0–111.5)	1585.000 (.977)^c^
Mechanical ventilation, days median (IQR)	47.0 (33.0–70.0)	45.0 (30.0–71.5)	50.5 (33.5–69.8)	1405.500 (.298)^c^
Number of medical comorbidities, median (IQR)	9.0 (7.0–12.0)	10.0 (8.0–13.0)	8.0 (6.3–11.0)	1098.000 (.005**)^c^
Psychological variables at (post-acute) ICU				
Perceived fear of dying at ICU^h^, median (IQR)	1.0 (1.0–6.0)	2.0 (1.0–6.0)	1.0 (1.0–5.0)	1375.500 (.321)^c^
Perceived social support according to MSPSS^h^, median (IQR)	6.3 (5.4–6.9)	6.3 (5.4–6.9)	6.3 (5.8–7.0)	1448.500 (.425)^c^
Diagnosis of major depression according to SCID I^h^, *n* (%)	9 (8.0)	8 (13.1)	1 (1.9)	5.023 (.035*)^f^
Diagnosis of posttraumatic stress disorder (PTSD) according to SCID I, *n* (%)	18 (15.9)	13 (21.3)	5 (9.6)	3.137 (.077)^d^
Prior psychiatric history				
History of harmful alcohol consumption, *n* (%)	22 (19.5)	12 (19.7)	10 (19.2)	.003 (.953)^d^
History of anxiety disorders, *n* (%)	8 (7.1)	5 (8.2)	3 (5.8)	.251 (.724)^f^
History of depressive disorders, *n* (%)	23 (20.4)	11 (18.0)	12 (23.1)	.441 (.507)^d^
History of psychological disorder, *n* (%)	70 (61.9)	34 (55.7)	36 (69.2)	2.168 (.141)^d^

*IQR* interquartile range, *MSPSS* Multidimensional Scale of Perceived Social Support, *PTSD* posttraumatic stress disorder, *SCID I* Structured Clinical Interview according to DSM IV

*p ≤ .05, **p ≤ .01

^a^Subsamples were generated using the cutoff score 53+ suggested by Kuhnt et al. [32]

^b^Statistical value and *p* value refer to the comparison between the subsamples of patients with high vs. low fatigue

^c^*p* value from Mann-Whitney *U* test

^d^*p* value from chi-squared test

^e^*n* = 6 missing values; high fatigue: *n* = 5, low fatigue: *n* = 1

^f^*p* value from Fisher’s exact test

^g^*n* = 1 brain, *n* = 5 central venous catheter, *n* = 1 port system, *n* = 1 urinary catheter; high fatigue: *n* = 1 brain, *n* = 1 central venous catheter, low fatigue: *n* = 1 port system, *n* = 1 urinary catheter, *n* = 4 central venous catheter

^h^*n* = 2 missing values

At t2, CCI patients with high fatigue showed a significantly lower Barthel index at discharge from rehabilitation hospital, a higher number of medical comorbidities, lived more often in a partnership and had more often a current diagnosis of major depression than patients with low fatigue (see Table 1 for detailed information). Patients with high fatigue more often showed certain medical comorbidities (e.g., left heart failure, coronary heart disease, organic brain syndrome, Additional file 1: Table S1). At t3, CCI patients with high fatigue were significantly older and had more often a diagnosis of major depression, posttraumatic stress disorder (PTSD), or prior history of an anxiety disorder (Additional file 2: Table S2).

### Point prevalence rates and intensity of fatigue

**Fig. 2:**
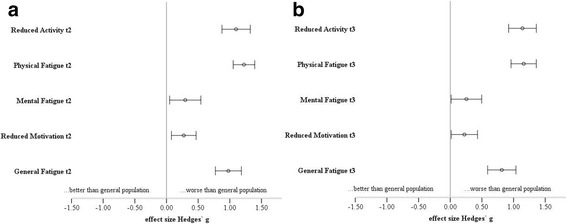
**a**, **b** Effect sizes with 95% CI comparing patients and the general population at t2 and t3. CI: confidence interval; German general population (*N* = 2037) according to Schwarz et al. [23]

According to the total score/the subscale General Fatigue of the MFI, 54.0% (*n* = 61)/46.9% (*n* = 53) and 49.5% (*n* = 45)/45.1% (*n* = 41) presented with clinically relevant symptoms of self-reported fatigue at t2 and t3, respectively. There was no significant difference with respect to the classification between t2 and t3 (McNemar test: *χ*^2^ = .000, *p* > .824). At both time points and in all MFI subscales, CCI patients reported fatigue scores about one standard deviation above the scores of a German general population [23]. High effect sizes were obtained for the MFI subscales Physical Fatigue, Reduced Activity, and General Fatigue (see Fig. 2a, b).

### Course of fatigue from 3 (t2) to 6 (t3) months following acute care ICU

**Fig. 3:**
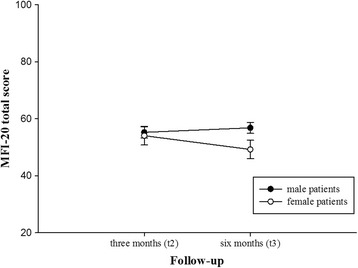
The course of the MFI-20 total score for male and female patients at 3 (t2) and 6 (t3) months following the discharge from ICU at acute care hospital. MFI-20: Multidimensional Fatigue Inventory

According to the *total fatigue* score, there was a significant time × gender effect (*F*(1, 88.0) = 5.604, *p* = .020, *η*^2^ = .060). While female patients showed a significant decrease over time, men remained stable from t2 to t3 (see Fig. 3). All other effects were non-significant (main effect of time: *F*(1, 88.0) = 1.262, *p* = .264, *η*^2^ = .014; main effect age: *F*(1, 88.0) = 2.749, *p* = .101, *η*^2^ = .030; main effect gender: *F*(1, 88.0) = 1.537, *p* = .218, *η*^2^ = .017; time × age: *F*(1, 88.0) = .010, *p* = .920, *η*^2^ = .000). Results were similar for the subscale General Fatigue. With respect to the other MFI subscales, there was no change from t2 to t3 (all *p* values > .512).

### Risk factors of fatigue in CCI patients

In univariate regression analyses, older age and living in a partnership were significant sociodemographic correlates of an increased fatigue score at t2. As clinical factors, the Barthel index at discharge from the rehabilitation hospital, the number of medical comorbidities, and the presence of a coronary heart disease could be identified. A SCID-I diagnosis of major depression or PTSD, the perceived fear of dying, and the perceived social support could be elucidated as significant psychological factors (Additional file 3: Table S3). In the multivariable regression model, controlling for age and gender, the presence of a coronary heart disease, the perceived fear of dying at ICU, a diagnosis of major depression, and the perceived social support explained 20.1% of the total variance (Table 2).

**Table 2:** Multivariable linear regression (stepwise) showing significant clinical and psychological variables of total fatigue as measured with the MFI-20 in chronically critically ill patients (*n* = 113) 3 months following the discharge from ICU at acute care hospital. The final model was controlled for age and gender

Multivariable linear regression^a^			
	Beta	CI	*p* value
Clinical variables			
Coronary heart disease	.27	.23–1.03	.002**
Psychological variables at (post-acute) ICU			
Perceived fear of dying at ICU	.25	.08–.42	.005**
Psychological variables 3 months following ICU			
Diagnosis of major depression according to SCID I	.26	.31–1.56	.004**
Perceived social support according to MSPSS	− .18	− .35–(−).01	.043*
*R*^2^ (corrected): .201 (*F*(4, 108) = 7.771, *p* < .001)			

^a^Method stepwise; PTSD at t2 and perceived fear of dying at ICU were significantly correlated (point biserial coefficient = .242, *p* = .011); family status and MSPSS at t2 were significantly correlated (point biserial coefficient = − .264, *p* = .005). Number of medical comorbidities and diagnosis of major depression/coronary heart disease were significantly correlated (point biserial coefficient = .279, *p* = .003/.305, *p* = .001). For parsimony of the final model and to prevent multicollinearity, PTSD at t2, family status and number of medical diagnoses were not considered in the final model. Tolerance/variance inflation factor and condition number test did not indicate multicollinearity

*MFI-20* Multidimensional Fatigue Inventory, *MSPSS* Multidimensional Scale of Perceived Social Support, *PTSD* posttraumatic stress disorder, *SCID I* Structured Clinical Interview according to DSM IV

*p ≤ .05, **p ≤ .01

At t3, partnership, female gender, a diagnosis of major depression/PTSD, the number of medical comorbidities, and a prior history of an anxiety disorder were significant correlates of an increased fatigue score (Additional file 4: Table S4). In a multivariable regression analysis, these factors explained a total variance of 31.8% (Table 3).

**Table 3:** Multivariable linear regression (stepwise) showing significant sociodemographic, clinical, and psychological variables of total fatigue as measured with the MFI-20 in chronically critically ill patients (*N* = 91) 6 months following the discharge from ICU at acute care hospital. The final model was controlled for age and gender

Multivariable linear regression^a^			
	Beta	CI	*p* value
Sociodemographic variables			
Gender (male vs. female)	− .23	− .91–(−).11	.013*
Clinical variables			
Number of medical comorbidities	.18	.00–.35	.045*
Psychological variables 6 months following ICU			
Diagnosis of major depression according to SCID I	.44	.80–1.87	< .001**
Prior psychiatric history			
History of anxiety disorder	.32	.55–1.85	< .001**
*R*^2^ (corrected): .318 (*F*(4, 90) = 11.497, *p* < .001)			

^a^Method stepwise; number of medical comorbidities and PTSD at t3/family status were significantly correlated (point biserial coefficient = .251, *p* = .016/.380, *p* < .001). For parsimony of the final model and to prevent multicollinearity, PTSD at t3 and family status were not considered in the final model. Tolerance/variance inflation factor and condition number test did not indicate multicollinearity

*MFI-20* Multidimensional Fatigue Inventory, *SCID I* Structured Clinical Interview according to DSM IV

*p ≤ .05, **p ≤ .001

### Association with posttraumatic stress and health-related quality of life

The total fatigue score was significantly positively correlated with the posttraumatic symptom score of the PTSS-10 at both t2 (Spearman’s rho = .656, *p* < .001) and t3 (Spearman’s rho = .600, *p* < .001). Fatigue was significantly negatively correlated with the health-related quality of life as measured using the EQ-5D-3L (t2: Spearman’s rho = − .648, *p* < .001; t3: Spearman’s rho = − .663, *p* < .001).

## Discussion

The main aim of the present study was to assess the frequency and the course of fatigue in CCI patients following protracted treatment on ICU. Furthermore, the investigation of related sociodemographic, clinical, and psychological factors was of interest in these patients. Currently, no study exists characterizing CCI patients with respect to a chronic state of exhaustion as aftermath following the survival of long-term mechanical ventilation. The present study finding is of clinical relevance, since fatigue is one of the most debilitating and distressing complaint in survivors of intensive care treatment [9, 18–20]. Unrecognized and untreated fatigue may have impeding effects on the patients’ health-related quality of life and rehabilitation process [16].

The findings of the present study elucidate that nearly every second patient suffered from an overwhelming sense of tiredness at rest up to 6 months post-ICU. This is in line with Steenbergen et al. [18] and Chaboyer and Grace [20] showing reports of clinically relevant fatigue between 37% and more than 50% even 1 year after ICU discharge. Moreover, CCI patients presented with fatigue scores one standard deviation above a representative sample of the adult German population which is in accordance with findings by Rosendahl et al. [45] in patients surviving severe sepsis.

According to the impact of gender on self-reported fatigue in CCI patients, female patients had lower values and presented a decline up to 6 months post-ICU. This is in contrast to other findings in the general population (e.g., [23, 46]). In the present sample, male patients were more severely ill than female patients as shown in a higher number of medical comorbidities (Mann-Whitney *U* = 934.500, *p* = .030). Thus, the symptoms of multiple concomitant diseases and the necessitating intensified treatment options may have led to a higher vital exhaustion in male CCI patients. However, our result should be interpreted thoughtfully, taking into account the peculiarities of the present sample; for instance, three quarter were male CCI patients. Nonetheless, gender differences in self-reported fatigue have not been consistently shown in the previous literature either (e.g., [47]). Beyond, in the present study, fatigue seemed to improve over time in female CCI patients whereas men showed a rather stable course. Former results pointed out that people affected by chronic fatigue improve with time but most remain functionally impaired for several years, independent from gender [48]. The course of fatigue has not yet been investigated before in CCI patients. Future studies should therefore more differentially consider the multiple aspects of fatigue in men and women suffering from CCI. Following, our result needs replication in a larger sample of CCI patients with an even distribution of male/female patients and a higher representation of patients 40 years or younger.

Regarding the impact of age, older CCI patients showed significantly higher fatigue values than younger CCI patients, particularly at t2. This result is consistent with data based on a German general population [23]. Increasing age goes along with a reduction in muscle strength, sarcopenia, and an increasing variability in motor neuron firing rates [2]. Above, the risk for chronic diseases, multi-morbidity, and a loss of psychosocial functioning increases with age and might thus contribute to higher fatigue scores [46].

A higher number of medical comorbidities and greater illness severity were associated with a higher self-reported fatigue. This is in line with the findings by Chlan and Savik [49] in mechanically ventilated patients. One may assume that more severely ill patients are exposed to an intensified medical therapy, an increased number of medications, and a heightened burden of concomitant diseases, entailing an increased fatigue level.

Three months post-ICU, the presence of a coronary heart disease turned out as one of the most prominent risk factor for increased fatigue values. Fatigue has been widely studied in patients with acute or chronic cardiovascular diseases (e.g., see [50, 51]). In these patients, fatigue constitutes one of the most distressing health complaints. It goes along with the inability to perform physical or intellectual efforts, a decreased health-related quality of life, and impedes participation in physical activity [51]. In the present sample of CCI patients, one quarter was affected by a chronic coronary heart disease (CHD). In these patients, an intricate interplay between neuroendocrine and hemodynamic dysfunction may lead to a mismatch of cardiac output to the patient’s need during exercise and goes along with peripheral muscle deconditioning. Together with the CIP/CIM as complication of critical illnesses such as sepsis, systemic inflammatory response syndrome, and multiple organ failure, difficulties in weaning from mechanical ventilation occurred in these patients and consequently led to prolonged ICU stays (median 47.0), long-term disabilities, and a muscular/cardio-respiratory deconditioning [52].

The perceived fear of dying at ICU, a SCID-based diagnosis of major depression or PTSD, and a prior anxiety disorder were identified as further prominent risk factors for increased self-reported fatigue in CCI patients. Moreover, fatigue levels were significantly related to posttraumatic symptomatology 3 and 6 months post-ICU. According to the DSM-IV/V, fatigue shows considerable conceptual overlap with some mood and anxiety disorders [24, 53, 54]. For instance, core features of a major depression are a loss of energy/vitality nearly every day, anhedonia, psychomotor retardation, and diminished ability to concentrate. These symptoms are also comprised in the MFI subscales, especially Reduced Activity/Reduced Motivation and Mental Fatigue. In line, Bunevicius et al. [51] showed that self-reported fatigue is strongly related to symptoms of anxiety and depression in patients with coronary artery disease. Above, Eckhardt et al. [7] have confirmed this result, proving depressive symptoms as sole predictor of fatigue intensity/interference from fatigue in patients with coronary heart disease.

Moreover, the fear of dying has not yet been proven as risk factor for increased fatigue in CCI patients. In a study by Wade et al. [55], the acute psychological reaction in the ICU displayed the strongest risk factor for poor psychosocial outcomes after ICU treatment. It can be supposed that patients with higher perceived fear of dying experienced a higher number of traumatic interventions because of a higher severity of medical illness. A correlational analysis with the Barthel index confirmed this assumption only by trend (Spearman’s *r* = − .172, *p* = .071). Otherwise, the peri-ICU stress reaction shares common features with anxiety and mood disorders and may constitute an indicator of a psychological vulnerability or a prior mental disorder predisposing the patient to the development of specific subfacets of fatigue (e.g., Mental Fatigue, Reduced Activity, Reduced Motivation) [7]. Furthermore, a shared pathophysiologic mechanism between affective disorders and fatigue with respect to the autonomous nervous system, the hypothalamic-pituitary-adrenal axis, or immunological functions has been demonstrated [56].

While the perceived social support turned out to be an essential resource and seems to have a buffering effect on self-reported fatigue in former studies (e.g., [46]), being married or living in a partnership could be identified as a risk factor for increased self-reported fatigue values in our present study. Patients living in a partnership do not necessarily receive greater social support than patients living alone. Survivors of CCI show profound changes in their family/friend relationships and their ability to participate in social roles and activities [8]. In our sample of CCI patients, partners were often affected by a chronic disease or disabilities themselves, keeping them from visiting the patient in the study center or granting adequate relief. Future studies should also implement a multidimensional measure of perceived social support and not solely rely on the simple assessment of partnership status via yes/no answering. Above, our finding of a salutogenetic impact of social support by family, friends, or significant others led us to conclude that a multidisciplinary approach is recommended in the treatment of self-reported fatigue in CCI patients, taking into account the needs of the patient’s whole social or family system.

An interesting and new finding of our present study displays the fact that major depression was the only common factor associated with fatigue at both t2 and t3. One reason for this might be the composition and selection of variables considered in the final models. For instance, some variables were excluded for reasons of parsimony of the final model and multicollinearity. Otherwise, there is evidence that certain variables have short-term effects on psychiatric outcomes like anxiety or depression while others, particularly related to continuing behaviors associated with prior psychiatric history, have long-term effects [57].

The results of our present study should be thoroughly evaluated in the context of methodological limitations. Generally, univariate analyses should be interpreted cautiously because they may be due to confounding. Above, the present study was primarily designed to assess a posttraumatic stress disorder in CCI patients following prolonged treatment on ICU. Fatigue was measured as secondary outcome. Nonetheless, a post hoc power analysis with Cohen’s *f*^2^ of .25 and .47 for t2 and t3, respectively; an *α* level of 5%; a sample size of *n* = 113/91; and eight regressors revealed a power of nearly 100%.

Another shortcoming concerns the lacking application of objective laboratory tests in order to validate the self-reported fatigue scores. Future studies should additionally determine exercise capacity measuring peak VO_2_, e.g., during bicycle ergometry, in order to specify the self-reported fatigue on an objective level and to differentiate between the physical and mental components of fatigue [50]. Also, the characterization of the functional status by measuring the activity of daily living and neuropsychological functioning should be realized in future studies. Furthermore, the impact of the current general medical conditions (e.g., cardiac, pulmonary, hepatic, renal, physical) as well as medication (e.g., sedatives, analgetics; [49]) on long-term fatigue intensity following protracted ICU treatment should be considered.

The validity of the point prevalence rates of fatigue reported in our present study has to be carefully considered since the use of the 75th percentile as cutoff criteria for high fatigue seems to be a bit arbitrary. The cutoff value for the total fatigue score was retrieved from a sample of cancer patients, not the general population [32]. When a more conservative cut-off criterion (90th percentile) for the classification of high fatigue is applied, after all, only *n* = 34 (30.1%) of the CCI patients could be still identified as cases at t2 and *n* = 22 (24.2%) at t3.

Another limiting fact concerns the mixture of patients without and with sepsis. The latter displays one of the most prominent risk factors for the development of CIP or CIM [52]. Mixing up both subsamples may have led to an inadequate estimation of the point prevalence rate of fatigue. We have decided to merge both subsamples because the validity of the sepsis diagnoses made by the acute care hospitals was questionable. Above, both subsamples did not significantly differ with respect to the main descriptive characteristics. In line, our results could be, by and large, replicated when only patients with sepsis were considered.

No information regarding the self-reported fatigue was available in the forefront of the ICU admission. Thus, we cannot causatively attribute high fatigue levels to the protracted treatment on ICU. Furthermore, patients of the present sample reported multiple reasons for fatigue; among them were morbidity, difficulties in breathing, medication, sleep disorders, and pain. Thus, profound diagnostics of fatigue are required leading to individually tailored interventions which are targeted on the multiple reasons of fatigue.

Finally, the present study sample of CCI patients can be regarded as a selective sample due to the high dropout rate of nearly 58%. Although the latter mirrors the daily clinical situation and is in line with other studies (e.g., [58]), it cannot be ruled out that the present fatigue values might be underestimated since more severely ill patients or patients with a lower functional status have not been followed up.

## Conclusion

To conclude, self-reported fatigue is a common symptom in CCI patients surviving intensive care after prolonged invasive ventilation. Male gender, the illness severity, a diagnosis of coronary heart disease, major depression, and the fear of dying at ICU were most intimately related to increased fatigue while the perceived social support turned out as salutogenetic factor. ICU survivors at risk should be regularly evaluated in routine clinical care following ICU discharge, and specialized interventions should be offered to them.

## Additional files

Additional file 1: **Table S1.** Medical comorbidities of chronically critically ill (CCI) patients (*n* = 113) and the subsamples of patients with high (*n* = 61) vs. low fatigue (*n* = 52) at 3 months (t2) following the discharge from ICU at acute care hospital. ^a^*p* value from chi-squared test; ^b^*p* value from Fisher’s exact test; *p ≤ .05, **p ≤ .01. (DOCX 18 kb)

Additional file 2: **Table S2.** Descriptive characteristics of chronically critically ill (CCI) patients (*n* = 91) and the subsamples of patients with high fatigue (*n* = 45) vs. low fatigue (*n* = 46) at 6 months (t2) following the discharge from ICU at acute care hospital. ^a^Subsamples were generated using the cutoff score 53+ suggested by Kuhnt et al. [32]; ^b^Statistical value and *p* value refer to the comparison between the subsamples of patients with high fatigue vs. low fatigue; ^c^*p* value from Mann-Whitney *U* test; ^d^*p* value from chi-squared test; ^e^*n* = 5 missing values; high fatigue: *n* = 2, low fatigue: *n* = 3; ^f^*p* value from Fisher’s exact test; ^g^*n* = 1 brain, *n* = 3 central venous catheter, *n* = 1 urinary catheter; high fatigue: *n* = 1 brain, *n* = 1 central venous catheter, low fatigue: *n* = 1 urinary catheter, *n* = 2 central venous catheter; IQR = interquartile range, *p ≤ .05. (DOCX 22 kb)

Additional file 3: **Table S3.** Univariate linear regression for the identification of sociodemographic, clinical, and psychological predictors of total fatigue as measured with the MFI-20 in chronically critically ill patients (*N* = 113) 3 months following the discharge from ICU at acute care hospital. ^1^*n* = 7 missing values; ^2^*n* = 1 missing value; ^3^*n* = 2 missing values; ASDS = Acute Stress Disorder Scale; ASD = Acute Stress Disorder; CAM-ICU = Confusion Assessment Method for the Intensive Care Unit; MFI-20 = Multidimensional Fatigue Inventory; MSPSS = Multidimensional Scale of Perceived Social Support; PTSD = Posttraumatic Stress Disorder; SCID I = Structured Clinical Interview according to DSM IV; *p ≤ .05, **p ≤ .01, ***p ≤ .001. (DOCX 18 kb)

Additional file 4: **Table S4.** Univariate linear regression for the identification of sociodemographic, clinical, and psychological predictors of total fatigue as measured with the MFI-20 in chronically critically ill patients (*N* = 91) 6 months following the discharge from ICU at acute care hospital. ^1^*n* = 7 missing values; ^2^*n* = 1 missing value; ^3^*n* = 2 missing values; ASDS = Acute Stress Disorder Scale; ASD = Acute Stress Disorder; CAM-ICU = Confusion Assessment Method for the Intensive Care Unit; MFI-20 = Multidimensional Fatigue Inventory; MSPSS = Multidimensional Scale of Perceived Social Support; PTSD = Posttraumatic Stress Disorder; SCID I = Structured Clinical Interview according to DSM IV; *p ≤ .05, **p ≤ .01, ***p ≤ .001. (DOCX 18 kb)

## Abbreviations

CAM-ICU: Confusion assessment method for the intensive care unit
CCI: Chronic critical illness
CIM: Critical illness myopathy
CIP: Critical illness polyneuropathy
DSM-IV: Diagnostic and Statistical Manual of Mental Disorders
EQ-5D-3L: Euro-Quality of Life
ICU: Intensive care unit
MFI-20: Multidimensional fatigue inventory
MSPSS: Multidimensional Scale of Perceived Social Support
PTSS-10: Posttraumatic Symptom Scale
SCID: Structured Clinical Interview for the Diagnostic and Statistical Manual of Mental Disorders DSM-IV
