# Supplementary material for: Self-reported fatigue following intensive care of chronically critically ill patients: a prospective cohort study
Source: J Intensive Care. 2018 May 2;6:27. doi: 10.1186/s40560-018-0295-7 (PMC5930426; doi:10.1186/s40560-018-0295-7)
Supplement: Supplementary file 1 — Table S1. Medical comorbidities of chronically critically ill (CCI) patients (n = 113) and the subsamples of patients with high (n = 61) vs. low fatigue (n = 52) at 3 months (t2) following the discharge from ICU at acute care hospital. ap value from chi-squared test; bp value from Fisher’s exact test; *p ≤ .05, **p ≤ .01. (DOCX 18 kb) [file 40560_2018_295_MOESM1_ESM.docx]

**Table S1:** Medical comorbidities of chronically critically ill (CCI) patients (n = 113) and the subsamples of patients with high (n = 61) vs. low fatigue (n = 52) at three months (t2) following the discharge from ICU at acute care hospital.

| **Characteristic** | **Patients followed-up**  **n = 113** | **High fatigue (n = 61)**^a^ | **Low fatigue (n = 52)**^a^ | **χ² *(p)***^b^ |
| --- | --- | --- | --- | --- |
| **Medical comorbidity** |  |  |  |  |
| **Lung** |  |  |  |  |
| Chronic obstructive pulmonary disease (COPD) (J44.X) | 31 (27.4) | 15 (24.6) | 16 (30.8) | .538 (.463)^a^ |
| Acute respiratory insufficiency (J96.00, J96.01, J96.09) | 87 (77.0) | 49 (80.3) | 38 (73.1) | .833 (.361)^a^ |
| Chronic respiratory insufficiency (J96.10,  J96.11, J96.19) | 10 (8.8) | 2 (3.3) | 8 (15.4) | 5.100 (.042*)^b^ |
| Pneumonia (J15,J18,J69) | 20 (17.7) | 12 (19.7) | 8 (15.4) | .354 (.552)^a^ |
| Sleep apnea (G47.3) | 14 (12.4) | 8 (13.1) | 6 (11.5) | .064 (.800)^a^ |
| **Diseases of the circulatory/ cardiovascular system** |  |  |  |  |
| Left heart failure (I50.1) | 40 (35.4) | 29 (47.5) | 11 (21.2) | 8.547 (.003**) ^a^ |
| Atrial fibrillation (I48.0-I48.2) | 39 (34.5) | 25 (41.0) | 14 (26.9) | 2.455 (.117)^a^ |
| Hypertension (I10.X) | 14 (12.4) | 40 (65.6) | 28 (53.8) | 1.611 (.204) |
| Coronary heart disease (I25.1) | 30 (26.5) | 23 (37.7) | 7 (13.5) | 8.461 (.004**) ^a^ |
| **Kidney** |  |  |  |  |
| Chronic kidney disease (N18.X) | 28 (24.8) | 16 (26.2) | 12 (23.1) | .150 (.699)^a^ |
| Urinary tract infection (N39.0) | 24 (21.2) | 13 (21.3) | 11 (21.2) | .000 (.984)^a^ |
| **Other** |  |  |  |  |
| Diabetes (E11.90) | 43 (38.1) | 27 (44.3) | 16 (30.8) | 2.168 (.141)^a^ |
| Adipositas (E66.X) | 28 (24.8) | 15 (24.6) | 13 (25.0) | .003 (.960)^a^ |
| Encephalopathy (G93.4) | 27 (23.9) | 17 (27.9) | 10 (19.2) | 1.152 (.283)^a^ |
| Organic brain syndrome (F06.9) | 44 (38.9) | 31 (50.8) | 13 (25.0) | 7.871 (.005**)^a^ |
| Neurological disorders | 27 (23.9) | 21 (34.4) | 6 (11.5) | 8.086 (.004**)^a^ |
| Cirrhosis of the liver | 2 (1.8) | 2 (3.3) | 0 (0.0) | 1.736 (.499)^b^ |
| Hypothyroidism | 22 (19.5) | 12 (19.7) | 10 (19.2) | .003 (.953)^a^ |

^a^*p*-value from Chi-squared test; ^b^*p*-value from Fisher´s exact test; *p≤.05, **≤.01
