# Supplementary material for: Self-reported fatigue following intensive care of chronically critically ill patients: a prospective cohort study
Source: J Intensive Care. 2018 May 2;6:27. doi: 10.1186/s40560-018-0295-7 (PMC5930426; doi:10.1186/s40560-018-0295-7)
Supplement: Supplementary file 2 — Table S2. Descriptive characteristics of chronically critically ill (CCI) patients (n = 91) and the subsamples of patients with high fatigue (n = 45) vs. low fatigue (n = 46) at 6 months (t2) following the discharge from ICU at acute care hospital. aSubsamples were generated using the cutoff score 53+ suggested by Kuhnt et al. [32]; bStatistical value and p value refer to the comparison between the subsamples of patients with high fatigue vs. low fatigue; cp value from Mann-Whitney U test; dp value from chi-squared test; en = 5 missing values; high fatigue: n = 2, low fatigue: n = 3; fp value from Fisher’s exact test; gn = 1 brain, n = 3 central venous catheter, n = 1 urinary catheter; high fatigue: n = 1 brain, n = 1 central venous catheter, low fatigue: n = 1 urinary catheter, n = 2 central venous catheter; IQR = interquartile range, *p ≤ .05. (DOCX 22 kb) [file 40560_2018_295_MOESM2_ESM.docx]

**Table S2:** Descriptive characteristics of chronically critically ill (CCI) patients (n = 91) and the subsamples of patients with high fatigue (n = 45) vs. low fatigue (n = 46) at six months (t2) following the discharge from ICU at acute care hospital.

| **Characteristic** | **Patients**  **n = 91** | **High fatigue (n = 45)**^a^ | **Low fatigue (n = 46)**^a^ | **U/ χ² *(p)***^b^ |
| --- | --- | --- | --- | --- |
| **Sociodemographic variables** |  |  |  |  |
| age, yrs median (IQR) | 60.0 (54.7-63.6) | 62.0 (55.5-65.6) | 58.0 (53.6-60.9) | 769.000 (.035*)^c^ |
| Gender, n (%) |  |  |  |  |
| Male | 67 (73.6) | 36 (80.0) | 31 (67.4) |  |
| Female | 24 (26.4) | 9 (20.0) | 15 (32.6) | 1.862 (.172)^d^ |
| Family status, n (%) |  |  |  |  |
| Single | 10 (11.0) | 5 (11.1) | 5 (10.9) |  |
| Married/cohabited | 60 (65.9) | 35 (77.8) | 25 (54.4) |  |
| Divorced/ living apart | 15 (16.5) | 3 (6.7) | 12 (26.1) |  |
| Widowed | 6 (6.6) | 2 (4.4) | 4 (8.7) | 8.200 (.085)^d^ |
| Partnership |  |  |  |  |
| yes | 78 (69.0) | 35 (77.8) | 25 (54.3) |  |
| no | 35 (31.0) | 10 (22.2) | 21 (45.7) | 5.560 (.018*)^d^ |
| Education, n (%)^e^ |  |  |  |  |
| < 10 yrs | 27 (29.7) | 14 (31.1) | 13 (28.3) |  |
| ≥ 10 yrs | 59 (64.8) | 29 (64.4) | 30 (65.2) | .054 (.816)^d^ |
| **Clinical variables** |  |  |  |  |
| Sepsis, n (%) |  |  |  |  |
| No sepsis | 29 (31.9) | 14 (31.1) | 15 (32.6) |  |
| Sepsis | 34 (37.4) | 19 (42.2) | 15 (32.6) |  |
| Severe sepsis or septic shock | 28 (30.8) | 12 (26.7) | 16 (34.8) | 1.066 (.587)^d^ |
| Number of sepsis episodes, median (IQR) | 1.0 (0.0-1.0) | 1.0 (0.0-1.0) | 1.0 (0.0-1.3) | 1016.000 (.871) ^d^ |
| Site of infection, n (%) |  |  |  |  |
| Respiratory | 47 (51.6) | 21 (46.7) | 26 (56.5) | .885 (.347)^d^ |
| Urinary/ genitals | 11 (12.1) | 6 (13.3) | 5 (10.9) | .130 (.718)^d^ |
| Abdominal | 7 (7.7) | 5 (11.1) | 2 (4.3) | 1.465 (.267)^f^ |
| Bones/ soft tissue | 5 (5.5) | 3 (6.7) | 2 (4.3) | .236 (.677)^f^ |
| Wound infection | 2 (2.2) | 1 (2.2) | 1 (2.2) | .000 (.987)^f^ |
| Heart | 1 (1.1) | 1 (2.2) | 0 (0.0) | 1.034 (.495)^f^ |
| Multiple | 12 (13.2) | 6 (13.3) | 6 (13.0) | .002 (.967)^d^ |
| Others^g^ | 5 (5.5) | 2 (4.4) | 3 (6.5) | .189 (1.000)^f^ |
| Unknown | 2 (2.2) | 1 (2.2) | 1 (2.2) | .000 (1.000)^f^ |
| Barthel index, median (IQR) |  |  |  |  |
| at admission at post-acute ICU | -195.0 (-225.0- -100.0) | -200.0  (-225.0- -100.0) | -170.0  (-225- -117.5) | 939.000 (.440)^c^ |
| at discharge from post-acute ICU | -30.0 (-80.0-10.0) | -25.0  (-80.0-20.0) | -35.0  (-76.3-6.3) | 1001.000 (.787)^c^ |
| at discharge from rehabilitation hospital | 70.0 (40.0-85.0) | 70.0  (10.0-85.0) | 75.0  (48.8-86.3) | 895.500 (.267)^c^ |
| ICU stay, days median (IQR) | 68.0 (49.0-96.0) | 74.0 (47.5-98.0) | 62.0 (50.5-90.0) | 970.500 (.609)^c^ |
| Mechanical ventilation, days median (IQR) | 47.0 (33.0-70.0) | 46.0 (30.0-76.0) | 50.0 (33.0-69.3) | 1026.000 (.943)^c^ |
| Number of medical comorbidities, median (IQR) | 9.0 (7.0-12.0) | 9.0 (7.5-12.0) | 8.5 (6.8-11.3) | 877.500 (.209)^c^ |
| **Psychological variables at (post-acute) ICU** |  |  |  |  |
| perceived fear of dying at ICU^h^, median (IQR) | 1.0 (1.0-6.0) | 3.0 (1.0-8.0) | 1.0 (1.0-5.0) | 753.000 (.037*)^c^ |
| Perceived social support according to MSPSS^h^, median (IQR) | 6.3 (5.6-6.8) | 6.3 (5.6-6.7) | 6.4 (5.6-6.9) | 910.500 (.321)^c^ |
| Diagnosis of Major Depression according to SCID I^h^, n (%) | 11 (12.1) | 10 (22.2) | 1 (2.2) | 8.604 (.004**)^f^ |
| Diagnosis of posttraumatic stress disorder (PTSD) according to SCID I, n (%) | 20 (22.0) | 18 (40.0) | 2 (4.3) | 16.861 (<.001***)^f^ |
| **Prior psychiatric history** |  |  |  |  |
| History of alcohol consumption, n (%) | 16 (17.6) | 8 (17.8) | 8 (17.4) | .002 (.961)^d^ |
| History of anxiety disorder, n (%) | 7 (7.7) | 7 (15.6) | 0 (0.0) | 7.752 (.006**)^f^ |
| History of depression, n (%) | 18 (19.8) | 8 (17.8) | 10 (21.7) | .225 (.635)^d^ |
| History of psychological disorder, n (%) | 56 (61.5) | 27 (60.0) | 29 (63.0) | .089 (.765)^d^ |

^a^subsamples were generated using the cut-off score 53+ suggested by Kuhnt et al. [32]; ^b^statistical value and p-value refer to the comparison between the subsamples of patients with high fatigue vs. low fatigue; ^c^*p*-value from Mann-Whitney-U test; ^d^*p*-value from Chi-squared test ; ^e^n = 5 missing values; high fatigue: n = 2, low fatigue: n = 3; ^f^*p*-value from Fisher´s exact test; ^g^n = 1 brain, n = 3 central venous catheter, n = 1 urinary catheter; high fatigue: n = 1 brain, n = 1 central venous catheter, low fatigue: n = 1 urinary catheter, n = 2 central venous catheter; IQR = interquartile range, *p≤.05
