# Supplementary material for: Self-reported fatigue following intensive care of chronically critically ill patients: a prospective cohort study
Source: J Intensive Care. 2018 May 2;6:27. doi: 10.1186/s40560-018-0295-7 (PMC5930426; doi:10.1186/s40560-018-0295-7)
Supplement: Supplementary file 3 — Table S3. Univariate linear regression for the identification of sociodemographic, clinical, and psychological predictors of total fatigue as measured with the MFI-20 in chronically critically ill patients (N = 113) 3 months following the discharge from ICU at acute care hospital. 1n = 7 missing values; 2n = 1 missing value; 3n = 2 missing values; ASDS = Acute Stress Disorder Scale; ASD = Acute Stress Disorder; CAM-ICU = Confusion Assessment Method for the Intensive Care Unit; MFI-20 = Multidimensional Fatigue Inventory; MSPSS = Multidimensional Scale of Perceived Social Support; PTSD = Posttraumatic Stress Disorder; SCID I = Structured Clinical Interview according to DSM IV; *p ≤ .05, **p ≤ .01, ***p ≤ .001. (DOCX 18 kb) [file 40560_2018_295_MOESM3_ESM.docx]

**Table S3:** Univariate linear regression for the identification of sociodemographic, clinical and psychological predictors of total fatigue as measured with the MFI-20 in chronically critically ill patients (N = 113) three months following the discharge from ICU at acute care hospital.

| **Univariate linear regression** | | | |
| --- | --- | --- | --- |
|  | **Beta** | **CI** | **P value** |
| **Sociodemographic variables** |  |  |  |
| Age | .18 | -.01-.36 | .059 |
| Gender | .02 | -.38-.46 | .845 |
| Family status, no partnership vs. partnership | .22 | .07-.86 | .021* |
| Education status < 10 yrs vs. ≥ 10 yrs^1^ | .01 | -.39-.44 | .909 |
| **Clinical variables** |  |  |  |
| sepsis, yes vs. no | .09 | -.20-.60 | .325 |
| Kind of sepsis |  |  |  |
| Sepsis | .14 | -.10-.67 | .139 |
| Septic shock | .03 | -.52-.70 | .773 |
| Severe sepsis | -.08 | -.66 -.26 | .393 |
| Number of sepsis episodes | .03 | -.16-2.14 | .786 |
| Barthel index at admission at post-acute ICU | .08 | -.11-.27 | *.*405 |
| Barthel index at discharge from post-acute ICU | .11 | -.08-.30 | .251 |
| Barthel index at discharge from rehabilitation hospital | -.21 | -.40-  (-).03 | .025* |
| Length of mechanical ventilation | -.06 | -.25-.13 | .521 |
| Length of ICU stay | -.04 | -.23-.15 | .656 |
| **Medical diagnosis** |  |  |  |
| Diabetes | .14 | -.10-.66 | .147 |
| Chronic kidney disease | -.04 | -.52-.35 | .704 |
| COPD | .02 | -.37-.47 | .826 |
| Hypothyreodism | .02 | -.43-.51 | .863 |
| Coronary heart disease | .24 | .12-.95 | .011* |
| Number of medical comorbidities | .33 | .15-.51 | <.001*** |
| **Psychological variables at (post-acute) ICU** |  |  |  |
| CAM-ICU sum score | .02 | -.17-.21 | .853 |
| perceived helplessness at ICU^2^ | .10 | -.09-.29 | .285 |
| perceived fear of dying at ICU^3^ | .24 | .05-.42 | .013* |
| Symptoms of Acute Stress Disorder (ASD) according to the ASDS^3^ | .08 | -.11-.27 | .415 |
| Diagnosis of ASD according to SCID I^3^ | .13 | -.19-.95 | .187 |
| Recalled experience of a traumatic event at ICU | .08 | -.24-.62 | .386 |
| **Psychological variables three months following post-acute ICU** |  |  |  |
| Perceived social support according to MSPSS^3^ | -.20 | -.38--.01 | .038* |
| Diagnosis of Major Depression according to SCID I^3^ | .27 | .34-1.68 | .004** |
| Diagnosis of posttraumatic stress disorder (PTSD) according to SCID I^3^ | .29 | .28-1.27 | .002** |
| **Prior psychiatric history** |  |  |  |
| History of depressive disorder | -.01 | -.49-.44 | .902 |
| History of harmful alcohol consumption | .00 | -.47-.47 | .999 |
| History of anxiety disorder | .14 | -.17-1.28 | .131 |

^1 n = 7 missing values; 2 n = 1 missing value; 3 n = 2 missing values; ASDS = Acute Stress Disorder Scale; ASD = Acute Stress Disorder; CAM-ICU = Confusion Assessment Method for the Intensive Care Unit; MFI-20 = Multidimensional Fatigue Inventory; MSPSS = Multidimensional Scale of Perceived Social Support; PTSD = Posttraumatic Stress Disorder; SCID I = Structured Clinical Interview according to DSM IV; *p ≤ .05, **p≤ .01, ***p ≤ .001^
