# Supplementary material for: Self-reported fatigue following intensive care of chronically critically ill patients: a prospective cohort study
Source: J Intensive Care. 2018 May 2;6:27. doi: 10.1186/s40560-018-0295-7 (PMC5930426; doi:10.1186/s40560-018-0295-7)
Supplement: Supplementary file 4 — Table S4. Univariate linear regression for the identification of sociodemographic, clinical, and psychological predictors of total fatigue as measured with the MFI-20 in chronically critically ill patients (N = 91) 6 months following the discharge from ICU at acute care hospital. 1n = 7 missing values; 2n = 1 missing value; 3n = 2 missing values; ASDS = Acute Stress Disorder Scale; ASD = Acute Stress Disorder; CAM-ICU = Confusion Assessment Method for the Intensive Care Unit; MFI-20 = Multidimensional Fatigue Inventory; MSPSS = Multidimensional Scale of Perceived Social Support; PTSD = Posttraumatic Stress Disorder; SCID I = Structured Clinical Interview according to DSM IV; *p ≤ .05, **p ≤ .01, ***p ≤ .001. (DOCX 18 kb) [file 40560_2018_295_MOESM4_ESM.docx]

**Table S4:** Univariate linear regression for the identification of sociodemographic, clinical and psychological predictors of total fatigue as measured with the MFI-20 in chronically critically ill patients (N = 91) six months following the discharge from ICU at acute care hospital.

| **Univariate linear regression** | | | |
| --- | --- | --- | --- |
|  | **Beta** | **CI** | **P value** |
| **Sociodemographic variables** |  |  |  |
| Age | .14 | -.07-.35 | .195 |
| Gender (male vs. female) | -.19 | -.90-.03 | .066 |
| Family status, no partnership vs. partnership | .21 | .01-.88 | .044* |
| Education status < 10 yrs vs. ≥ 10 yrs^1^ | -.03 | -.53-.42 | .813 |
| **Clinical variables** |  |  |  |
| sepsis, yes vs. no | .04 | -.37-.53 | .731 |
| Kind of sepsis |  |  |  |
| Sepsis | .06 | -.30-.56 | .550 |
| Septic shock | .00 | -.66-.68 | .986 |
| Severe sepsis | -.04 | -.62 -.44 | .736 |
| Number of sepsis episodes | .01 | -.21-.22 | .958 |
| Barthel index at admission at post-acute ICU | -.07 | -.28-.15 | *.*539 |
| Barthel index at discharge from rehabilitation hospital | -.19 | -.40-.01 | .066 |
| Length of mechanical ventilation | .05 | -.17-.26 | .669 |
| Length of ICU stay | .13 | -.08-.34 | .215 |
| **Medical diagnosis** |  |  |  |
| Diabetes | .13 | -.15-.70 | .204 |
| Chronic kidney disease | -.02 | -.54-.45 | .852 |
| COPD | -.05 | -.59-.38 | .667 |
| Hypothyreodism | -.03 | -.58-.45 | .792 |
| Coronary heart disease | .04 | -.40-.60 | .689 |
| Number of medical comorbidities | .24 | .03-.44 | .023* |
| **Psychological variables at (post-acute) ICU** |  |  |  |
| CAM-ICU sum score | .01 | -.20-.22 | .925 |
| perceived helplessness at ICU^2^ | .13 | -.08-.34 | .222 |
| perceived fear of dying at ICU^3^ | .20 | -.01-.41 | .064 |
| Symptoms of Acute Stress Disorder (ASD) according to the ASDS^3^ | .11 | -.11-.32 | .320 |
| Diagnosis of ASD according to SCID I^3^ | .17 | -.12-1.10 | .114 |
| Recalled experience of a traumatic event at ICU | .07 | -.32-.62 | .529 |
| **Psychological variables six months following post-acute ICU** |  |  |  |
| Perceived social support according to MSPSS^3^ | -.17 | -.37-.04 | .116 |
| Diagnosis of Major Depression according to SCID I | .37 | .54-1.74 | <.001*** |
| Diagnosis of PTSD according to SCID I | .37 | .52-1.72 | <.001*** |
| **Prior psychiatric history** |  |  |  |
| History of depressive disorder | -.03 | -.61-.45 | .761 |
| History of harmful alcohol consumption | -.00 | -.56-.55 | .986 |
| History of anxiety disorder | .31 | .40-1.89 | .003** |

^1 n = 7 missing values; 2 n = 1 missing value; 3 n = 2 missing values; ASDS = Acute Stress Disorder Scale; ASD = Acute Stress Disorder; CAM-ICU = Confusion Assessment Method for the Intensive Care Unit; MFI-20 = Multidimensional Fatigue^ ^Inventory; MSPSS = Multidimensional Scale of Perceived Social Support; PTSD = Posttraumatic Stress Disorder; SCID I = Structured Clinical Interview according to DSM IV; *p ≤ .05, **p≤ .01, ***p ≤ .001^
